# Molecular Analysis of Thymoma

**DOI:** 10.1371/journal.pone.0042669

**Published:** 2012-08-13

**Authors:** Sunil Badve, Chirayu Goswami, Yesim Gökmen–Polar, Robert P. Nelson, John Henley, Nick Miller, Narjis A. Zaheer, George W. Sledge, Lang Li, Kenneth A. Kesler, Patrick J. Loehrer

**Affiliations:** 1 Department of Pathology and Laboratory Medicine, Indiana University School of Medicine, Indianapolis, Indiana, United States of America; 2 Department of Medicine, Indiana University School of Medicine, Indianapolis, Indiana, United States of America; 3 Center for Computational Biology and Bioinformatics, Indiana University School of Medicine, Indianapolis, Indiana, United States of America; 4 Department of Surgery, Indiana University School of Medicine, Indianapolis, Indiana, United States of America; 5 Indiana University Melvin and Bren Simon Cancer Center, Indianapolis, Indiana, United States of America; 6 Columbus Regional Hospital, Columbus, Indiana, United States of America; University Medical Centre Utrecht, The Netherlands

## Abstract

Histologic classification of thymomas has significant limitations with respect to both subtype definitions and consistency. In order to better understand the biology of the disease processes, we performed whole genome gene expression analysis. RNA was extracted from fresh frozen tumors from 34 patients with thymomas and followup data was available. Using the Illumina BeadStudio® platform and Human Ref-8 Beadchip, gene expression data was analyzed with Partek Genomics Suite®, and Ingenuity Pathways Analysis (IPA). Unsupervised clustering of gene expression data, representing one of the largest series in literature, resulted in identification of four molecular clusters of tumors (C1–C4), which correlated with histology (*P* = 0.002). However, neither histology nor clusters correlated with clinical outcomes. Correlation of gene expression data with clinical data showed that a number of genes were associated with either advanced stage at diagnosis or development of recurrence or metastases. The top pathways associated with metastases were amino acid metabolisms, biosynthesis of steroids and glycosphingolipids, cell cycle checkpoint proteins and Notch signaling. The differential expression of some of the top genes related to both metastases and stage was confirmed by RT-PCR in all cases of metastases and matched nonmetastatic cases. A number of potential candidates for therapeutics were also identified.

## Introduction

Thymic epithelial neoplasms are rare with the incidence of thymic carcinoma being around 0.2% to 1.5% of all malignancies [1], [2]. Although rare, these neoplasms are associated with controversies, which initially revolved around the inclusion of nonepithelial tumors such as lymphomas and germ cells tumors in this category [3]. The histological classification remains controversial, partly because thymic neoplasms tend to be slow growing tumors, and complete surgical excision can be curative irrespective of the histological features [4]–[6]. In an effort to standardize the classification for these tumors, the World Health Organization (WHO) in 1999 devised consensus categories [7]. As of 2004, the following major categories are recognized: type A, AB, B1, B2, B3, and thymic carcinoma [8] (type C in 1999 classification [7]).

A number of factors including presence of myasthenic symptoms, tumor size, completeness of surgical excision in addition to tumor histology, and stage have been associated with the likelihood of recurrence. However, the data for most of these factors comes from small studies due to rarity of disease and lack of large randomized trials. In order to progress beyond clinical, operative, and morphological features, we analyzed the gene expression pattern of these tumors and correlated them with outcomes in a relatively large series of patients.

## Methods

### Patient samples

Following Indiana University Institutional Review Board (IRB) approval, all patients with thymic neoplasms were (written) consented for tissue collection for research use. Additional IRB approval was specifically obtained for use of clinical data and for performing gene expression analysis on all cases that had fresh frozen thymic tumors available at the Indiana University Simon Cancer Center Tumor Bank. It is a standard practice at this institution to divide the tissue procured for banking and generate a paraffin block of quality control. A histology review of the cases was performed on the paraffin counterpart of the frozen tissues by a single pathologist (SB); cases of thymic carcinoma and thymoma type A (*n* = 1 each) were excluded. This retrospective review identified 34 cases of thymomas, which were further categorized based on predominant pattern according to the 2004 WHO classification [8]. The tumors were categorized into three groups: group I (*n* = 9), type AB; group II (*n* = 19), types B1–B2; and group III (*n* = 6), type B3. Importantly in order to understand the role of tumor microenvironment including lymphocytic component, microdissection was not performed.

The clinical information including the followup data of the patients was acquired by review of the medical charts. The variables such as age, gender, type of surgery, complete or incomplete resection, histologic subtype, tumor stage, clinical features, and presence of autoimmune disease like myasthenia gravis were recorded. The tumors had been staged using the Masaoka staging system: stage 1 (no invasion of the capsule), stage 2 (focal invasion of the capsule by tumor or invasion of mediastinal fat), stage 3 (direct extension into adjacent structures such as pericardium, large vessels, or lung), and stage 4 (pleural, pericardial implants, or distant metastases) [9]. Details of chemotherapy and development of adverse events including relapse and metastasis were also recorded.

### Demographics

The clinical details of the 34 patients with thymic neoplasms included in the study are detailed in Table 1. The age range was 17 to 76 years with a mean of 52 years. Women were slightly more represented in the series with M∶F ratio of 1∶1.4.

**Table 1:** Clinical characteristics of the patients included in the gene expression analysis.

Characteristics		Data
**Age**	Mean	52 (17–76)
**Sex**	Male	14
	Female	20
**Tumor size mean (range)**		8.16 cm (0.6–25 cm)
**Histology**	AB	9
	B1–B2	19
	B3	6
**Stage**	I/II	22
	III/IV	12
**Paraneoplastic syndrome**	Positive	13
	Negative	21
**Chemotherapy**	Positive	10
	Negative	24
**Relapse**	Positive	8
	Negative	26
**Metastasis**	Positive	8
	Negative	26

### Clinical features

Most of the 34 patients presented with a mediastinal mass associated with local or paraneoplastic symptoms. Thirteen patients had associated paraneoplastic manifestations. Myasthenia gravis was seen in ten patients. Of these ten patients, lupus was present in one, while red cell aplasia and Guillain–Barré syndrome were observed in another patient. Of the three patients who are not associated with myasthenia gravis, one patient had Grave disease and another patient had Hashimoto syndrome. The third patient presented with multiple paraneoplastic features of pure red cell aplasia and hypogammaglobulinemia. At diagnosis, ten patients were stage I, 12 at stage II, six at stage III, and six at stage IV disease. Six patients were also identified to have a second malignancy (two ovarian cancers and one each of nonsmall cell lung cancer, glioblastoma, breast cancer, and prostate cancer).

### Treatment details

All patients had surgery with a curative intent. Of the 34 patients, ten received chemotherapy with carboplatin and paclitaxel based regimens. Eight patients did receive radiotherapy.

### Followup data

Eight patients relapsed with disease and eight developed metastases which were identified either at diagnosis or during the followup period. Histologically, these eight metastatic cases were subtyped as follows: two AB, three B1, one B2, and two B3. Twenty-six patients did not have relapse, while 26 patients had no evidence of metastases during the followup period. Histologically, the relapsed cases were of the following subtypes: one AB, two B1, two B2, and three B3.

### Microarray analysis

RNA was extracted using TRIzol® Reagent method (Invitrogen, Carlsbad, CA) according to the manufacturer's instructions. The quality of RNA was assessed using the Nanodrop® ND-1000 spectrophotometer (ThermoScientific, Wilmington, DE). Integrity was measured by calculating the RNA integrity number on the Agilent Bioanalyzer (Agilent Technologies, Santa Clara, CA). All RNA samples were treated with Turbo DNase® (Ambion/Applied Biosystems, Foster City, CA). To assess RNA performance further, real-time quantitative RT-PCR (qRT-PCR) analysis was performed for *RPL13A* according to Illumina's instructions (San Diego, CA). Following qualification, 200 ng of total RNA was used for whole genome – cDNA-mediated annealing, selection, extension, and ligation (WGDASL; Illumina, San Diego, CA) analysis as per manufacturer's protocol. The whole genome DASL Assay consists of modified RT-PCR reaction following which the product is captured on beads. The probe set used was the Illumina Human Ref-8 BeadChip. This BeadChip features up to date content covering more than 24,000 annotated genes derived from RefSeq (Build 36.2, Release 22).

## Analysis

### Data Preprocessing

Data on 34 patients from Illumina Human WGDASL arrays, with each array containing 18401 probes were analyzed. Genes which had a poor signal quality across a maximal number of arrays were filtered out. As a result, 8260 genes were found to have signals significantly above background. The samples were run in three batches and batch effect was removed statistically using Partek Genomics suite's batch effect removal tool. The data was quantile normalizedand log_2_ transformed before statistical analysis.

### Unsupervised clustering and differential gene expression analysis

Processed data was hierarchically clustered using Partek Genomics Suite with Pearson dissimilarity and average linkage as clustering parameters. We identified four major clusters from the hierarchical clustering results. We performed one way ANOVA analysis to identify differentially expressed genes in our dataset. ANOVA analysis was done for thymoma groups (GI, GII, and GIII), metastatic versus nonmetastatic groups, and stage I/II versus III/IV groups separately to identify differentially expressed genes in these comparisons.

### Ingenuity Pathways Analysis

To identify the statistically significant biological functions, and signaling pathways affected by the genes differentially expressed in our comparisons, we performed Ingenuity Pathways Analysis (IPA; Ingenuity Systems, Inc). IPA is the largest curated database and analysis system for understanding the signaling and metabolic pathways, molecular networks, and biological processes that are most significantly changed in a dataset of interest (http://www.ingenuity.com).

### Validation of the chosen genes by real-time quantitative RT-PCR

Based on ANOVA *P* value and fold change, we selected three top genes associated with metastasis YES phenotype for validation. All qRT-PCR reactions were performed in duplicate. Total RNAs were reverse-transcribed using high capacity cDNA reverse transcription kit. The mRNA levels were analyzed by real-time qRT-PCR using TaqMan® gene expression assays on an ABI Prism 7900 platform according to the manufacturer's instructions (Applied Biosystems, Foster City, CA). Importin 8 (*IPO8*) and transferrin receptor (*TFRC*) were used as endogenous controls for normalization purpose. These were selected from a panel of housekeeping genes using TaqMan human endogenous control array (Applied Biosystems), as they showed minimal variability in expression across the thymoma patients. Expression data of the target genes was normalized by using the geometric mean of *IPO8* and *TFRC*. The relative quantification of the gene expression changes (fold) was analyzed according to ΔΔCt method using the Applied Biosystems DataAssist™ Software v3.0.

## Results

### Unsupervised clustering

**Figure 1:**
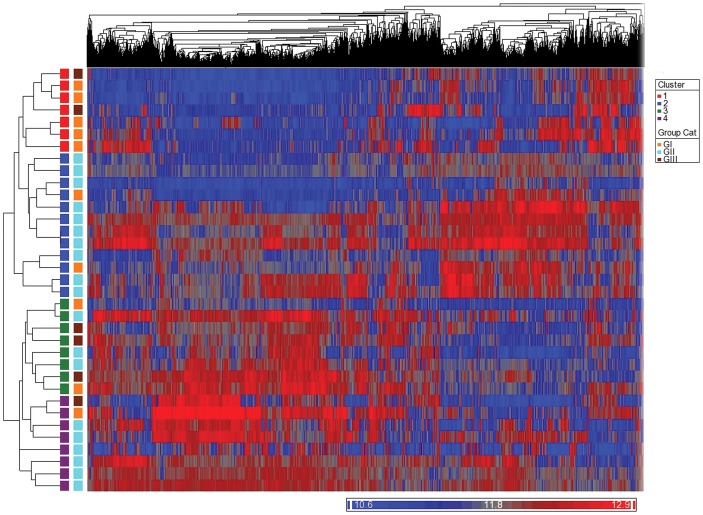
Unsupervised hierarchical clustering of the 34 fresh-frozen thymomas showing four distinct clusters (C1–C4). One sample is included as the duplicate.

Unsupervised cluster analysis revealed that the duplicate specimens, as expected, clustered together. Using the 8260 genes that were statistically significant for gene expression analysis (Figure 1), four distinct clusters (labeled C1–C4) composed of 6, 12, 8, and 8 tumors, respectively, were identified. None of the clusters was purely composed of tumors of any particular histologic subtype. Of the six tumors in cluster 1, four were of type AB tumors and two were of B3 type. Similarly, in cluster 2, ten were of type B1–B2 tumors; and two type AB tumors. Cluster 3 contained two type AB tumor, three type B1–B2 tumors, and three type B3 tumors. Similarly, cluster 4 was composed of six B1–B2 tumors, one type AB tumor, and one B3 tumors. The differential enrichment of clusters, with some clusters having samples belonging predominantly to one thymomas group, was statistically significant (*P* = 0.002; dof = 15).

### Supervised clustering

**Figure 2:**
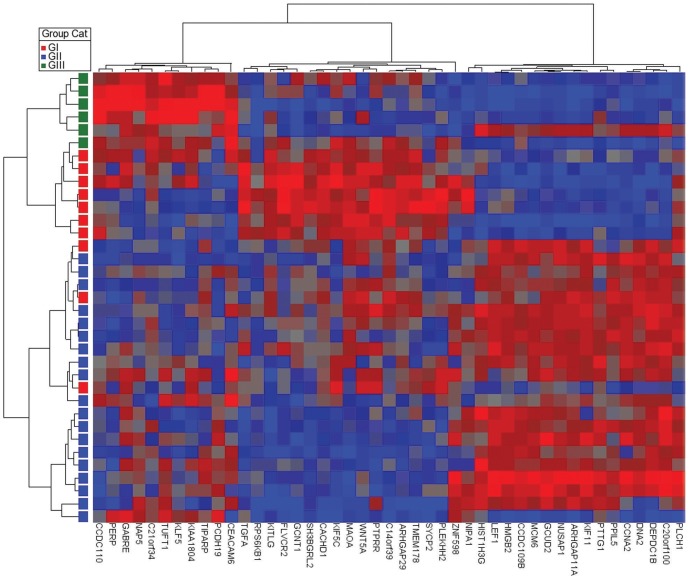
Supervised hierarchical clustering of the fresh-frozen thymomas based on the histologic groups. The figure comprises of 34 tumors (GI = group I (type AB), GII = group II (types B1–B2), and GIII = group III (type B3) and one duplicate.

On the basis of significant differential expression (*P*<0.01) with histological groups in ANOVA analysis, we selected top 15 upregulated (highest positive fold changes) and 15 downregulated genes (highest negative fold changes) to generate a supervised clustering heat map (Figure 2). The first group of 22 tumors was composed of 19 type B1–B2, and three type AB tumors. The second group of seven tumors was composed of six type AB tumors and one type B3 tumors. One of the AB tumors was performed in duplicate. The third group was composed of five B3 tumors. This data highlights the fact that by gene expression morphologically heterogeneous tumors may cluster together.

It is well observed that patients with thymomas have a tendency to develop second malignancies, predominantly AB thymomas [10]. In our study, five of group 1 (type AB) and one of group 2 (B2) were associated with second malignancies. Using 30 upregulated and 30 downregulated genes (ANOVA; *P*≤0.001), supervised analysis of tumors from patients with second malignancies versus without second malignancies identified two main clusters with various subsets (Figure S1). Despite the heterogeneity, tumors with second malignancy revealed a distinct subset associated with top biological functions such as infectious disease, dermatological disease, hereditary disorder protein synthesis, cell death, and nucleic acid metabolism (IPA analysis). Further confirmatory studies are required to understand the mechanistic aspect of the association of second malignancies with thymoma.

### Correlation of histology and gene expression clusters with metastases and stage

Among the 34 cases analyzed there was no correlation of histological type with stage of tumor at diagnosis or development of metastases. Similarly, the analysis of the association of the clusters with these clinical parameters did not show a correlation. Hence we did further analyses to identify genes that predict the development of metastases.

### Biological functions and canonical pathways associated with metastasis and stage of thymoma

**Figure 3:**
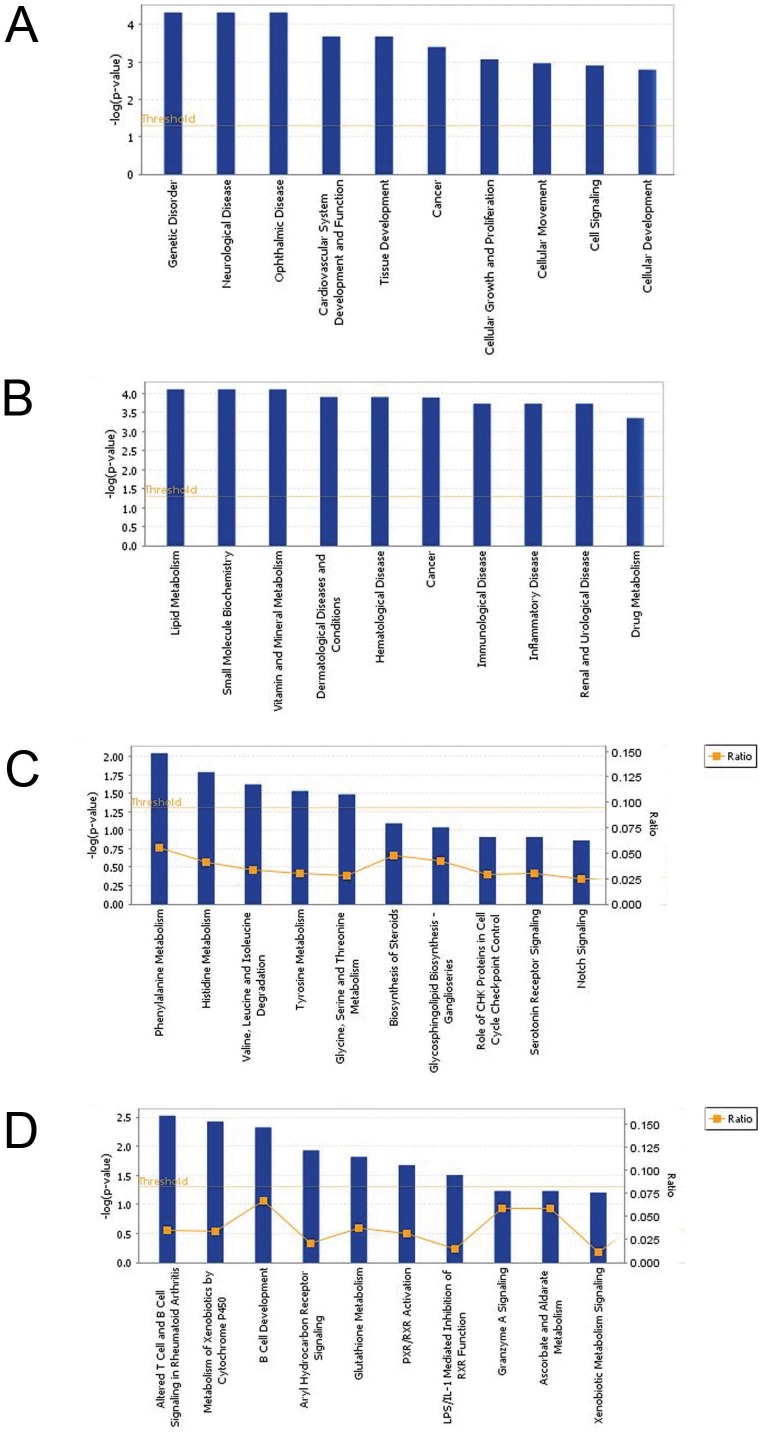
Biological processes and canonical pathways associated with metastasis and stage using Ingenuity Pathways Analysis. The top ten significant biological functions. (A) Metastasis, (B) stage, and canonical pathways. (C) Metastasis and (D) stage were grouped based on the *P* values using right tailed Fisher exact test and with threshold less than 0.05.

Using IPA analysis, we further explored the identification of the most significant biological functions associated with diseases at the molecular and cellular levels and pathways in the above samples associated with metastatic phenotype (Met Yes group) compared with nonmetastatic group (Met No group) (Figure 3). Figure 3A shows the top ten significant functions associated with metastatic phenotype. These included functions associated with genetic disorder, neurological disease, and cancer, and molecular and cellular functions such as cellular growth and proliferation, cellular and cellular development (Figure 3A). Similar analyses with respect to stage I/II compared with stage III/IV showed cancer, dermatological diseases and conditions, hematological diseases, immunological disease, inflammatory disease, lipid metabolism, vitamin and mineral metabolism, and drug metabolism (Figure 3B). Table S1 and Table S2 show detailed lists of top ten biological functions for metastasis and stage, respectively.

To further provide insight into signaling of the differentially expressed genes associated with clinical outcome, we next categorized genes associated with metastasis and stage into canonical pathways using IPA. The most significant pathways associated with metastasis were amino acid metabolisms including phenylalanine, histidine, and tyrosine (Figure 3C). Other pathways included biosynthesis of steroids, glycosphingolipid biosynthesis, role of CHK proteins in cell cycle checkpoint control, serotonin receptor signaling, and Notch signaling. In stage analysis, altered T cell and B cell signaling in rheumatoid arthritis, B cell development, aryl hydrocarbon receptor signaling, PXR/RXR activation, and LPS/IL-1 mediated inhibition of RXR function were among the top ten pathways (Figure 3D). Table S3 and Table S4 show detailed lists of top ten canonical pathways for metastasis and stage, respectively.

### Confirmation of the differential expression for the selected genes in clinical parameters

**Figure 4:**
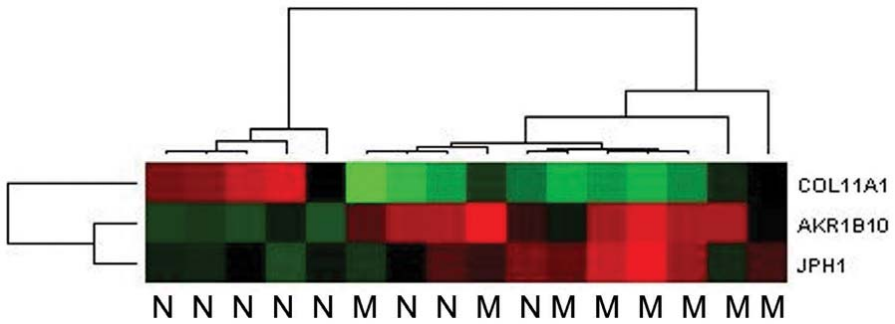
Heat map representing the mRNA levels of *AKR1B10*, *JPH1*, and *COL11A1* genes using TaqMan qPCR system. Data is normalized to the geometric mean of the reference genes *IPO8 and TFRC*. The heat map is generated using the Applied Biosystems DataAssist™ v3.0 software. M, tumors associated with metastasis, N, tumors with no metastasis.

To validate the differential expression of the top potential genes in relation to the clinical parameters such as metastasis, quantitative real-time RT-PCR (qRT-PCR) analysis was performed. The genes were verified in all metastatic samples (*n* = 8) and matched set of nonmetastatic tumor samples (*n* = 8). Among the top genes chosen, two of them (*AKR1B10 and JPH1*) were upregulated in both metastasis and stage III/IV categories (IPA), whereas one of them (*COL11A1*) was downregulated in both metastasis and stage III/IV categories. Relative mean expression of selected genes (as 2^−ΔCt^ values) in each set was calculated and used to determine the fold change of the metastatic group relative to the nonmetastatic group. Representative qRT-PCR heatmap for *AKR1B10*, JPH1, *and COL11A1* was shown in Figure 4. Briefly, *AKR1B10 and JPH1* showed a 5.96-fold (*P* = 0.05) and 3.01 (*P* = 0.18)-fold increase in the metastasis-positive tumors compared with the metastasis-negative tumors, respectively *COL11A1* was decreased 12.5-fold (*P* = 0.11) in metastasis-positive tumors.

## Discussion

Thymomas are enigmatic tumors in that their biology is poorly understood. Complete surgical excision seems to be the best predictor of behavior [11]. The role of histological (WHO) classification in predicting behavior of thymic tumors, apart from thymic carcinomas, is still controversial [5]. In this study, we attempted to understand the biology of these tumors using whole genome gene expression analysis. Although a few gene expression studies have been previously performed in thymic tumors [12]–[14], these have been handicapped by lack of clinical data. In the current study, we have followup information on most of the patients, which has permitted limited correlation of the gene expression findings with clinical behavior recognizing that these findings may be altered to some extent by surgical, radiation, and chemotherapy. It is accepted that these therapeutic interventions make the patient population being analyzed less than pristine and the observations noted in these studies have to be tempered. However, thymic tumors are rare, making this one of the largest series of fresh frozen tumors with followup information from patients published to date. In spite of the above limitations, the gene expression analysis revealed some very exciting results. These microarray results were further confirmed by quantitative RT-PCR for a number of genes.

The principal findings include a relatively good correlation of histology with gene expression (*P* = 0.003), in both unsupervised and supervised clustering. However, the current study and that performed by Girard et al [14] highlight the need for further refinement in the histological classification. In addition, neither histology nor gene expression associated clusters were associated with outcomes; ie, relapse or metastases.

Differential gene expression of a few hundred to over a thousand genes depending on the comparison was observed between histologic groups in these series (data not shown). Some of these genes corresponded to the loss of chromosomal regions that have been previously described [15]–[17]; eg, loss of Chr6p in type A–AB thymomas. In an aCGH (array comparative genomic hybridization) analysis of 39 thymomas, additional losses of Chr2, Chr4, and Chr13 were identified in type A thymomas, and Chr1q gain in B3 thymomas [13]. Loss of Chr2 in type A and gain in 1q in B3 was confirmed in our study. In this regard, it worth noting that a number of genes belong to the histone cluster in Chr1 was lost in A–AB group. Lee et al [13] identified 50 genes at an FDR of 1.7% that could distinguish between types A and B. Some of these genes (eg, *LEPR*) were similarly differentially expressed in our series and that of Girard et al [14]. Similarly, they identified 48 genes that could reliably distinguish between types B1–B2 and B3, three of these (*CDC2L5* LEPR and *LYPLAL1*) were also differentially expressed in our analysis.

In an analysis of 36 patients using an oligonucleotide array, Sasaki et al [12] identified 13 genes that had greater than twofold differential expressions. However, only *c-JUN* and AL050002 correlated with tumor stage by qRT-PCR and only AL050002 gene correlated with B3 histology; in the current study, expressed above background noise of the array (data not shown).

None of the prior studies has associated gene expression with tumor metastasis, stage, or relapse. A number of these genes were associated with adverse (B3) histological features, higher stage, and metastasis. Aldo-keto reductase family 1 B10 (*AKR1B10*) is an enzyme overexpressed in multiple cancers including lung, pancreas, liver, uterine, and breast cancer [18]–[23].

Overexpression of *COL11A1*, Hedgehog target gene, is associated with poor prognosis in lung cancer [24]. Very little is known about the role of junctophilin-1 (*JPH1*) in cancer. One study reported that *JPH1* was upregulated in the poor outcome group in neuroblastoma patients [25]. Based on the differential expression levels and association with cancer, these genes were selected for validation by qRT-PCR. These qRT-PCR studies confirmed that these genes were differentially expressed in the different phenotypes (Figure 4).

In summary, whole genome expression analysis identified four clusters of thymic tumors, which show significant correlation with histological classification. A number of genes associated with clinical behavior of thymic tumors including stage, relapse, and metastasis were identified, and some were confirmed using qRT-PCR. However, the data presented here still needs to be externally validated in independent cohort(s) of cases. With this aim in mind, we are in the process of collating archival formalin-fixed paraffin-embedded blocks from patients with thymomas treated at a number of institutions (in addition to ours). Confirmation of the data will be performed using immunohistochemical and multiplex quantitative RT-PCR methods using a candidate based approach. It is important to note that some of the genes that we have identified and confirmed by qRT-PCR (such as *AKR1B10* and *JPH1*) may serve as potential candidates for targeted therapeutics in cancer.

## Supporting Information

Figure S1 **Supervised clustering of patients with secondary malignancy (** ***n*** **= 6) and patients without secondary malignancy.** Heatmap was generated using the top 30 upregulated genes and the top 30 downregulated genes.
(TIF)

Table S1 **Top ten biological processes for metastasis.**
(XLS)

Table S2 **Top ten biological processes for stage.**
(XLS)

Table S3 **Top ten canonical pathways for metastasis.**
(XLS)

Table S4 **Top ten canonical pathways for stage.**
(XLS)
