# Supplementary figures and images for: Molecular Analysis of Thymoma
Source: PLoS One. 2012 Aug 13;7(8):e42669. doi: 10.1371/journal.pone.0042669 (PMC3418289; doi:10.1371/journal.pone.0042669)

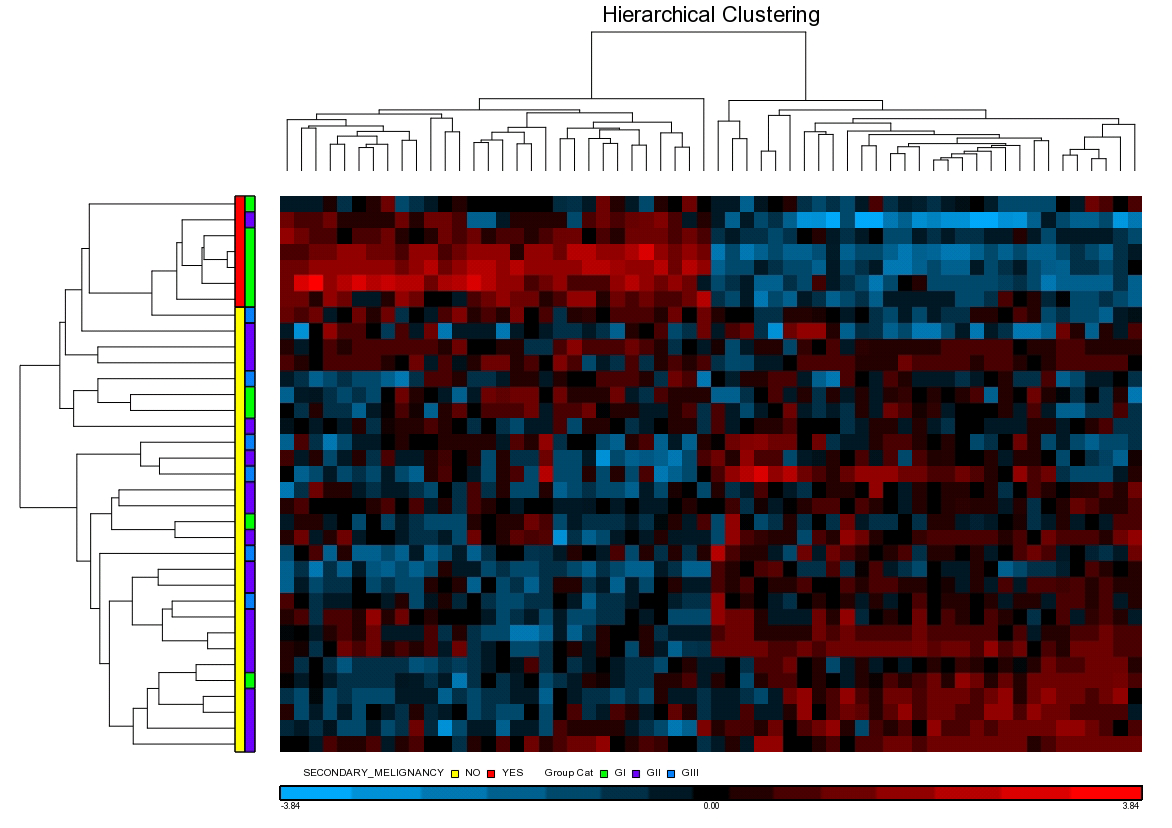

Supplement: Figure S1 — Supervised clustering of patients with secondary malignancy ( n = 6) and patients without secondary malignancy. Heatmap was generated using the top 30 upregulated genes and the top 30 downregulated genes. (TIF) [file pone.0042669.s001.tif]
